# Exploring the Influence of Oral‐Proximal Gastric in Vitro Digestion on Estimated Glycemic Index and Bioaccessibility of Bioactive Phenolic Compounds of Wheat‐Based Baked Products

**DOI:** 10.1002/fsn3.72014

**Published:** 2026-06-11

**Authors:** Jasper Okoro Godwin Elechi, Diana Marisol Abrego‐Guandique, Roberto Cannataro, Nicola Gasparre, Erika Cione

**Affiliations:** ^1^ Department of Pharmacy, Health, and Nutritional Sciences University of Calabria Arcavacata Rende Italy; ^2^ Department of Food and Human Nutritional Sciences University of Manitoba Winnipeg Canada; ^3^ Richardson Centre for Food Technology and Research University of Manitoba Winnipeg Canada; ^4^ Department of Health Sciences University of Magna Graecia Catanzaro Catanzaro Italy; ^5^ Galascreen Laboratories University of Calabria Rende Cosenza Italy; ^6^ Research Division Dynamical Business & Science Society, DBSS International SAS Bogota Colombia

**Keywords:** flavonoid release, glycemic index, in vitro digestion, phenolic bioaccessibility, salivary α‐amylase, starch hydrolysis, wheat‐based bakery products

## Abstract

Oral processing during the act of eating entails the cyclic mechanical breakdown and insalivation of food to create a bolus that is both safe and simple to ingest. This process is crucial for sensory perception, which in turn affects the blood glucose response through the enzymatic hydrolysis of starch. In vitro digestion studies frequently neglect the oral and proximal gastric phases, which are the sites of initiation and extension of α‐amylase exposure. Therefore, the objective of the current study was to investigate the impact of oral‐proximal gastric in vitro digestion on the glycemic response and phenolic compounds of bread and cookies. The findings indicated that the food particle size distribution during the 60‐min proximal gastric digestion was influenced by the extended salivary oral phase in the so‐called proximal gastric phase. This was achieved through the irregular generation and leaching of small particles (< 2 mm) through starch hydrolysis. The phenolics and flavonoids of bread and cookies were released to varying degrees of irregularity at various digestion stages and among the food samples. The proportion of sugar released increased swiftly in a nonlinear manner over the course of the 60 min, and all bread samples, irrespective of their initial starch and ingredient compositions, exhibited similar glucose release at the conclusion of the 60‐min oral‐proximal gastric digestion. The rates of starch digestion during the oral‐proximal gastric phase were found to be highly correlated with a widely used in vitro protocol for estimating the glycemic index of foods that attest to the significant contribution of prolonged human salivary α‐amylase activity in the oral and proximal gastric phase to Glycemic responses to starch‐based food. Therefore, the development of starch‐based foods that preserve organoleptic properties and effectively inhibit human salivary α‐amylase activities, regardless of the individual's production level, could be a viable approach to slowing down starch hydrolysis by Human Salivary Amylase (HSA) the enzyme and reducing the Glycemic index score.

## Graphical abstract

This study investigated how oral and proximal gastric digestion influences starch breakdown, estimated Glycemic index (eGI), and the bioaccessibility of phenolic compounds in wheat‐based baked products (bread and cookies). Simulated digestion showed that prolonged human salivary α‐amylase activity increased starch hydrolysis, glucose release, and the formation of small food particles during the first 60 min of digestion. Phenolic compounds and flavonoids were released at different rates depending on product type and digestion stage. Oral‐proximal gastric starch digestion was strongly associated with eGI. Incorporating this digestion phase into in vitro models improves prediction of Glycemic response and bioactive compound bioaccessibility in wheat‐based foods.

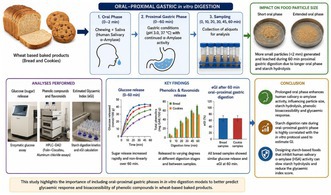

## 1 Introduction

The global rise in diet‐related disorders, such as obesity, type 2 diabetes, and cardiovascular disease, has intensified pressure on the food industry to deliver healthier products that meet consumer expectations for both taste and nutrition (Elechi et al. 2023). This shift has transformed food research priorities: while earlier efforts focused largely on sensory appeal, current studies emphasize the physiological and metabolic consequences of food consumption (Norton et al. 2014; Nadia et al. 2024). Understanding how food structure influences digestion, nutrient release, and bioactive compound bioaccessibility is therefore central to designing functional foods that contribute to long‐term health. Recent studies highlight that digestion is not merely a process of nutrient liberation but a critical determinant of metabolic health outcomes, including glycemic control and gut microbiota modulation (Wojtunik‐Kulesza et al. 2020; Somaratne et al. 2020).

One area of growing importance is the digestion of starch, the predominant dietary carbohydrate worldwide. Historical shifts in human diets following the advent of agriculture significantly increased starch intake, which coincided with evolutionary adaptations such as higher salivary amylase gene copy numbers (Peyrot Des Gachons and Breslin 2016). Despite this adaptation, many individuals with low salivary amylase activity continue to consume high‐starch diets, placing them at greater risk for metabolic syndrome and glucose dysregulation. Epidemiological evidence strongly links high Glycemic load diets to type 2 diabetes (Bhupathiraju et al. 2014), coronary heart disease (Dong et al. 2012), and obesity‐related complications (Schwingshackl and Hoffmann 2013). These findings underscore the need to investigate starch digestibility not only in terms of enzymatic hydrolysis but also in relation to postprandial glucose responses and the release of bioactive compounds.

Although starch digestion is initiated in the oral cavity through the action of human salivary α‐amylase (HSA), research has often underestimated this step due to the short residence time in the mouth. However, recent evidence demonstrates that HSA may remain active well beyond the oral phase, extending into the early gastric phase (Freitas et al. 2018; Freitas and Le Feunteun 2019; Nadia et al. 2022). This challenges the long‐held assumption that salivary amylase is immediately inactivated upon entering the stomach, where acidic conditions prevail (pH 1.5–3). In reality, gastric pH fluctuates dynamically after meals, rising temporarily to 5–6 before gradually returning to fasting levels (Bornhorst and Singh 2012; Freitas and Le Feunteun 2019). During this period, HSA remains active, continuing to hydrolyze starch and shaping the pool of digestible carbohydrates available for subsequent intestinal digestion.

Equally important is the spatial heterogeneity of gastric physiology. The proximal stomach acts as a temporary food reservoir with limited mixing, while the distal stomach is the primary site of acid secretion and mechanical breakdown (Nadia et al. 2022). These distinct environments influence starch hydrolysis differently, with extended contact between active salivary amylase and food in the proximal stomach enhancing early starch degradation (Freitas, Souchon, and Le Feunteun 2022; Gao et al. 2021). Furthermore, emerging evidence suggests that interactions between starch digestion and phytochemicals may modulate antioxidant bioaccessibility and Glycemic response, offering a more holistic view of food's impact on health (Tagle‐Freire et al. 2022; Wu et al. 2017). Despite these advances, most in vitro digestion models still rely on simplified static conditions that overlook proximal gastric dynamics, limiting their ability to replicate in vivo physiology.

Despite extensive research on gastrointestinal digestion, early oral and proximal gastric events remain underrepresented in conventional static in vitro digestion models, even though they play a critical role in determining subsequent nutrient release and metabolic response. In particular, the contribution of active human salivary *α‐amylase* (HSA) beyond the oral phase and its interaction with food structure require further clarification. Given the central role of starch‐rich foods in global diets and their contribution to dietary Glycemic load and metabolic disease risk, wheat‐based bakery products provide a highly relevant model system for investigating these early digestive dynamics. Their structural and compositional diversity makes them especially suitable for examining how early digestive dynamics influence starch hydrolysis and phytochemical bioaccessibility. Wheat bread and cookies were selected based on four key criteria: (i) high starch content and widespread consumption, ensuring public health relevance; (ii) variability in processing conditions known to influence starch gelatinization, retrogradation, and matrix architecture; (iii) structural diversity to examine differences in enzymatic accessibility; and (iv) practical applicability through the use of commercially available products. Therefore, three types of commercially available sliced wheat bread were included to capture variability in fermentation, baking temperature, and formulation, all of which substantially affect starch structure and digestibility. In contrast, a single oatmeal cookie was incorporated as a representative compact oat‐based product. Unlike bread, cookies are produced under more standardized industrial conditions with comparatively less structural variability across brands. Thus, the cookie was included primarily as a structural and compositional contrast to porous bread matrices, characterized by higher fat content and the presence of oat‐derived phenolic compounds, rather than as a within‐category comparison group.

Accordingly, this study aimed to evaluate the independent and interactive effects of prolonged oral‐proximal exposure to active HSA and food matrix composition on (i) food breakdown behavior and particle size reduction, and (ii) downstream nutritional outcomes including starch digestibility, reducing sugar and glucose release, phenolic compound bioaccessibility, and predicted Glycemic response. We hypothesized that sustained HSA activity during the proximal gastric phase would enhance structural disintegration and starch hydrolysis, thereby increasing sugar liberation and phenolic availability, and that matrix‐dependent structural characteristics would significantly modulate these effects. By explicitly linking early digestive phase dynamics to both physical transformation and metabolic prediction, this study refines static in vitro digestion modeling and provides mechanistic insight into how food structure and oral‐proximal interactions collectively shape nutrient bioaccessibility and simulated Glycemic outcomes.

## 2 Materials and Methods

### 2.1 Materials

#### 2.1.1 Food Samples

Wheat‐based baked products were selected based on their high starch content, widespread consumption, and structural diversity. Three commercially available wheat breads and one oatmeal cookies were purchased from a local Canadian supermarket (Real Canadian Superstore, Winnipeg, Canada). Products were classified according to grain composition and labeling information provided on packaging. The bread samples included 100% refined single‐grain bread (White Bread), 100% whole single‐grain bread (Whole Wheat Bread) and 100% whole multigrain bread (Whole Multigrain Bread). A commercially available oatmeal cookie was included as a structural contrast to wheat breads, rather than as a representative of all oat‐based bakery products. Ingredient lists and nutritional information, as provided by manufacturers, are presented in Table S1. Information regarding detailed processing conditions (e.g., baking time, temperature, moisture content, and fermentation parameters) was not available from manufacturers, and no independent structural or thermal characterization (e.g., DSC or XRD) was performed. Therefore, assumptions regarding variability in starch gelatinization and matrix architecture are based on differences in product type and formulation as indicated on product labeling, rather than confirmed processing data.

#### 2.1.2 Saliva Collection, Chemicals and Reagents

Unstimulated whole saliva was collected from 19 healthy volunteers (12 females, 7 males) with no history of gastrointestinal disorders, recent antibiotic use, or excessive alcohol consumption, following informed consent. Participants were instructed to refrain from eating, drinking, brushing teeth, chewing gum, or using tobacco products for at least 30 min prior to collection. Saliva samples were pooled to minimize interindividual variability, aliquoted into 50 mL tubes, and stored at −80°C to avoid repeated freeze–thaw cycles. No additional pretreatment was performed to preserve physiological composition, particularly endogenous salt content, which may influence HSA activity. Prior to digestion experiments, aliquots were thawed and equilibrated at 37°C for 5 min.

The following enzymes were used for in vitro digestion: Pepsin P7125 (porcine gastric mucosa; 679 U/mg protein), Pancreatic α‐amylase A‐3176 (porcine pancreas; 9.9 U/mg solid), and Amyloglucosidase A‐10113 (Aspergillus niger; ≥ 120 U/mg). All enzymes were obtained from Sigma‐Aldrich (St. Louis, MO, USA). The Total Starch Assay Kit (AA/AMG; K‐TSTA‐100A), Resistant Starch Assay Kit (K‐RAPRS), and D‐Glucose Assay Kit (GOPOD Format; K‐GLUC) were purchased from Megazyme (Wicklow, Ireland). All other chemicals and solvents were of analytical grade.

### 2.2 Experimental Design and Digestion Procedure

#### 2.2.1 Bread and Cookies Image Profile

The exterior image structures of the bread and cookie samples were obtained using a flatbed scanner. Each slice of bread and cookie was scanned on both sides via a flatbed scanner (CanoScan 9000F Mark II, Canon, USA) at a resolution of 600 dpi and stored as a polychrome picture.

#### 2.2.2 Food Preparation

A cooking knife was used to separate the bread crust from the crumb. All of the tests were done on the bread crumb. A KitchenAid coffee grinder was used to grind the bread and cookies so that moisture, starch, and phenolic chemicals could be measured.

#### 2.2.3 Moisture Contents and Nutritional Characterization of Bread and Cookies Samples

The moisture content of bread samples was measured in triplicate using a digital moisture analyzer (Denver Instrument IR‐35, Broomfield, USA.) by weighing 3 g of samples and heating them at 130°C for 30 min. The manufacturer's specified values for macro and micronutrients are included in the Table S1.

#### 2.2.4 Quantifications of Starch Content

The total and resistant starch contents were quantified in triplicate and utilized to estimate digestible starch. Total starch (TS) was quantified with the Total Starch test Megazyme kit from Megazyme (Bray, Ireland), adhering to the protocol for assessing total starch concentration in samples containing resistant starch. The rapid resistant starch kits test methodology from Megazyme (Bray, Ireland) was utilized to ascertain the resistant starch (RS) level. Digestible starch (DS) was determined by subtracting resistant starch from total starch. The acquired values, TS, DS, and RS, were quantified in grams of starch per 100 g of total starch on a wet basis.

#### 2.2.5 Extraction of Bioactive Compounds From Bread and Cookies

The extraction of bioactive chemicals was performed according to the method established by Najjar et al. (2022), as adapted by Pinto, Moreira, Vieira, et al. (2023). Crushed bread and cookies (1 g) were combined with 5 mL of distilled water, subjected to sonication at 40°C for 5 min, incubated at 4°C for 30 min, centrifuged at 10,000× g for 10 min, and the supernatant was thereafter collected. The solid residue was subsequently re‐extracted using 2 mL of distilled water, subjected to sonication at 40°C for 15 min, maintained at 4°C for 30 min, and centrifuged at 10,000× g for 10 min. The supernatants were subsequently collected and preserved at −20°C until analysis. The extraction was conducted in duplicate.

#### 2.2.6 Determination of Total Phenolic Compounds and Flavonoids

Total phenolic content (TPC) and total flavonoid content (TFC) were determined using extracts prepared from 1.0 g of homogenized sample (wet weight basis), as described in Section 2.2.5 (Extraction Procedure). Following extraction, the supernatant was collected and used for spectrophotometric analyses. All results were normalized to the initial sample mass and expressed on a wet weight basis (mg equivalents per g sample).

##### 2.2.6.1 Total Phenolic Content (TPC)

TPC was quantified using the Folin–Ciocalteu method (Singleton and Rossi 1965). A gallic acid standard solution was prepared by dissolving 50 mg of gallic acid in 1 mL ethanol and diluting to 10 mL with distilled water. Working standards (0, 50, 100, 250, and 500 mg/L) were prepared from this stock solution. For analysis, 20 μL of sample extract was mixed with 1.58 mL deionized water and 100 μL Folin–Ciocalteu reagent. After vortexing, 300 μL of 7% sodium carbonate was added, and the mixture was incubated at 40°C for 30 min. Absorbance was measured at 765 nm. TPC was calculated using the gallic acid calibration curve (*y* = 0.0009*x*, *R* ^2^ = 0.9999). Results were expressed as milligrams of gallic acid equivalents per gram of sample (mg GAE/g wet weight), taking into account dilution factors and the initial extraction mass.

##### 2.2.6.2 Total Flavonoid Content (TFC)

Total flavonoid content was determined using the aluminum chloride colorimetric method (Zhishen et al. 1999, with modifications). A quercetin stock solution (4 mg/mL) was prepared in methanol and serially diluted (0–10 mg/mL) for calibration. For analysis, 150 μL of sample extract was mixed with 25 μL methanol and 75 μL of 5% NaNO_2_ and allowed to react for 5 min at room temperature. Subsequently, 1.25 mL of 7% AlCl₃ and 0.5 mL of 5% NaOH were added. After centrifugation at 5000× g for 10 min and standing for 15 min, absorbance was measured at 510 nm. TFC was quantified using the quercetin calibration curve (*y* = 0.0007*x*, *R* ^2^ = 0.986). Results were expressed as milligrams of quercetin equivalents per gram of sample (mg QE/g wet weight), accounting for dilution factors and the original extraction mass.

### 2.3 Oral‐Proximal Gastric Static In Vitro Digestion Procedure

The digesting process used a shaking water bath, with conditions and duration tailored to mimic the movement of the food bolus in the oral‐proximal stomach. It comprised an oral phase, succeeded by a proximal gastric phase characterized by extended incubation in saliva postoral phase.

#### 2.3.1 Oral Phase

The technique established by Freitas and Le Feunteun (2019) was utilized for the oral phase. The oatmeal cookies and breadcrumbs were combined in a domestic blender for 2 s at level 1 or 5 s at level 2 to replicate food comminution akin to human mastication, without the addition of saliva at this step. The blending approach has been shown to provide boluses that most nearly mimic the physical features of in vivo masticated boluses (Gao et al. 2019). The 2 s and 5 s blending conditions were applied as standardized mechanical treatments to generate reproducible initial bolus structures and were not intended to replicate validated human mastication particle size distributions. The combined samples were stored in an airtight container at −80°C until used. Saliva was heated to 37°C in a hot‐air oven for 10 min immediately before each digesting experiment. Each saliva‐based bolus was performed by placing 2.5 g of artificially chewed samples into a separate 50 mL Corning‐Falcon container and mixing with 1.5 mL of saliva for 30 s using vortexing. This was subsequently incubated for 2 min in accordance with the INFOGEST methodology and recommendations for the oral phase (Brodkorb et al. 2019).

#### 2.3.2 Proximal Gastric Phase

Following the oral phase, the digesta was subjected to a prolonged incubation period referred to as the *proximal gastric phase* (Nadia et al. 2022). This phase was designed to mimic the physiological conditions of the proximal stomach, where ingested food initially resides and continues to interact with salivary enzymes before substantial acidification occurs. The concept is based on observations from studies in developing pigs, which demonstrated that the proximal region of the stomach maintains a relatively mild pH environment during the early stages of digestion (Bornhorst et al. 2014; Nadia et al. 2021). The in vitro protocol described by Nadia et al. (2022) was adapted to reproduce this phase. Specifically, the incubation time established during the oral phase was extended for 0 (no extension), 10, 15, 20, 30, 45, and 60 min. Each incubation time was conducted in a separate container to allow complete recovery of the digesta at the end of each digestion condition. To terminate enzymatic activity, the entire digestion mixture from each proximal gastric phase was immediately mixed with 0.4 mL of 6 M NaOH, raising the pH to approximately 10. This approach effectively halted further enzymatic reactions while preserving the physical characteristics of the digesta. The samples were then stored at −80°C until subsequent analyses.

### 2.4 Analysis of Food Digesta

The food digesta from Section 2.3.2 comprises a combination of digestive products and original food components at any stage of digestion (Bornhorst et al. 2016; Lu et al. 2021). In vitro digesta were characterized, and the effects of oral‐proximal stomach digestion on particle size, glucose, reducing sugar release from starch, bioaccessibility of phenolic compounds, and digestibility were assessed. The food digesta were thawed at 4°C and centrifuged (10 min, 3000× g, 4°C) to segregate the suspended fine particle fragments generated during food breakdown from the liquid medium containing digestive products. The fine particle pellets and supernatants were collected and stored at −80°C for further analysis.

#### 2.4.1 Dry Mass Pellet Particle Distribution

Food digesta consists of a dynamic mixture of partially degraded food structures and soluble digestive products at any stage of digestion (Bornhorst et al. 2016; Lu et al. 2021). In the present study, in vitro digesta were characterized to evaluate the effects of static oral‐proximal gastric digestion on structural breakdown, particle size distribution, starch‐derived glucose and reducing sugar release, phenolic compound bioaccessibility, and overall digestibility. The food digesta were thawed at 4°C and centrifuged (10 min, 3000× g, 4°C) to segregate the suspended fine particle fragments generated during food breakdown from the liquid medium containing digestive products. The fine particle pellets and supernatants were collected and stored at −80°C for further analysis.

Particle size distribution was not measured immediately after blending. Instead, structural disintegration was assessed following completion of the oral‐proximal digestion period to capture the combined effects of mechanical and enzymatic breakdown. Particle size distribution was subsequently determined following the methodology described by Gao et al. (2015). Fine particle pellets were immersed in 20 mL of 100% ethanol to facilitate separation of aggregated bolus fragments through gentle agitation. Ethanol was removed using Whatman filter paper, and the moistened particles were oven‐dried at 60°C for 30 min. Drying was performed solely to enable consistent sieving and quantification on a dry mass basis. Partially dried particles were then passed through a 2‐mm mesh sieve to separate coarse (> 2 mm) and fine (< 2 mm) fractions. The mass of retained and passed fractions was recorded. Dried particles larger than 2 mm were scanned using a flatbed scanner (CanoScan 9000F, Canon) to document pellet dimensions.

#### 2.4.2 Determination of Glucose and Starch Release

The digesta supernatants from Section 2.4 were defrosted at 4°C. Aliquots of supernatant (100 μL) were combined with 1.840 mL of sodium acetate buffer (0.4 M, pH 4.75) and incubated with 60 μL of *amyloglucosidase* (3300 U/mL) at 60°C for 45 min in a shaking water bath to achieve full hydrolysis of starch into glucose. The glucose liberated from starch breakdown in the samples at various time intervals was measured using the glucose oxidase‐peroxidase (GOP) reaction, utilizing a K‐GLUC enzymatic kit (Megazyme, Wicklow, Ireland). The concentration of free glucose was assessed by incubating the samples with the GOP reagent at 50°C for 20 min, followed by absorbance measurement using a spectrophotometer at 510 nm (Synergy HT, Biotek Instruments Inc., Winooski, VT). A glucose solution at a concentration of 1 mg/mL served as the standard. The analyses were conducted in triplicate for every sample and sampling period. The extent of starch hydrolysis was determined as the ratio of liberated glucose to sample weight.

#### 2.4.3 Quantifications of the Release of Reducing Sugars Using DNS Colourimetry

The digesta supernatants from Section 2.4 were defrosted at 4°C. The measurement of reducing sugars was performed by mixing 0.2 mL of digesta with 0.2 mL of dinitrosalicylic acid (DNS) working solution and heating the combination in a shaking water bath at 100°C for 15 min. The samples were then cooled to room temperature and diluted to 5 mL with distilled water. Three aliquots of 250 μL of the sample were dispensed into a 96‐well microplate, and the absorbance was quantified using a microplate reader (Synergy HT, Biotek Instruments Inc., Winooski, VT) at 540 nm. The reducing sugar was measured in maltose equivalents (mg maltose/g sample) using a maltose calibration curve represented by *y* = 0.0017*x* with *R* ^2^ = 0.9989, where y indicates absorbance, and *x* represents maltose concentration (mg/mL). The concentration of reducing sugar in the dietary digesta is shown against time.

#### 2.4.4 Recovery of Phenolic Compounds From Digested Samples

The total phenolic compounds and flavonoids in the digesta were quantified using the supernatants from Section 2.4, using the methodology described in Section 2.2.6. The phenolics recovery rate was determined using the method proposed by Pinto, Moreira, Švarc‐Gajić, et al. (2023), computed as the ratio of the phenolics recovered after each digestion period to the total phenolics present in the food sample. Results were computed as a percentage using equation (1):

(1) $$\text{Recovery}\;\left(\%\right)=\left(\text{PCDS}/\text{PCUS}\right)\times 100$$

where PCDS refers to the phenolic content of the digested sample and PCUS refers to the phenolic content of the undigested sample.

#### 2.4.5 Bioaccessibility of Phenolic Compounds

In the oral‐proximal gastric in vitro digestion, the bioaccessibility of each phenolic compound was estimated as the proportion of the compound released from the sample at each digestion stage compared to the undigested compound. Consequently, the recovery index following all digestive steps reflects the bioaccessibility (%) and was computed using the equations (2) and (3) shown below:

(2) $$\text{Oral Bioaccessibility}\;\left(\%\right)=\left(\text{Average of Oral fraction}/\text{Total phenolic content}\right)\times 100$$

(3) $$\text{Proximal gastric Bioaccessibility}\;\left(\%\right)=\left(\text{Average of proximal gastric fraction}/\text{Total phenolic content}\right)\times 100$$

### 2.5 In Vitro Starch Digestibility and Estimated Glycaemic Index

The GI of each product was assessed with the in vitro method outlined by Goñi et al. (1997). In summary, 100 mg samples of wheat bread and oatmeal cookies were duplicated, combined with 10 mL of 0.1 M KCl‐HCl buffer (pH 1.5), and incubated for 60 min at 40°C in a shaking water bath with 200 μL of pepsin solution (6790 U/mL; P‐7125, Sigma‐Aldrich Inc. St. Louis, Mo., U.S.A) prepared at 20 mg/2 mL KCl‐HCl buffer to replicate gastric proteolysis. 25 mL of 0.1 M TRIS‐maleate buffer (pH 6.9) was included to maintain a pH of 6.9, followed by incubation at 37°C for 180 min with 5 mL of pancreatic *α‐amylase* (A‐3176, Sigma‐Aldrich Inc. St. Louis, Mo., U.S.A) solution (3 U/mL in TRIS‐maleate buffer) to replicate intestine amylolysis. During the second incubation, 1 mL samples were taken in triplicate at 0, 5, 10, 20, 30, 40, 50, 60, 75, 90, 105, 120, 150, and 180 min, and subsequently added to 1 mL of 96% ethanol to inactivate the enzymatic reaction, followed by refrigeration at 4°C until the conclusion of the incubation period (180 min). All samples were maintained at 4°C for 24 h prior to analysis according to the methodology outlined in Section 2.4.2 for glucose and released starch. Partially hydrolysed starch was determined as mg of glucose multiplied by 0.9.

#### 2.5.1 Rapidly and Slowly Digested Starch

Rapidly digestible starch (RDS) and slowly digestible starch (SDS) were quantified following the methodology established by Englyst et al. (1992). The enzymatic hydrolysis technique employed by Goñi et al. (1997) was utilized to get these fractions. In this study, RDS denotes any starch that is digested early in the small intestine phase, regardless of its physical shape, and generally adheres to the RDS definition established by Englyst et al. (1992). RDS was evaluated as the ratio of starch transformed into reducing sugars after 20 min of amylolytic digestion, whereas SDS was determined as the percentage of starch digested after 120 min.

#### 2.5.2 Hydrolysis Index and Estimated Glycemic Index

The digestion curves were fitted using the nonlinear equation established by Goñi et al. (1997) to characterize the kinetics of starch hydrolysis Equation (4):

(4) $$C=C_{\infty }\;\left(1-e^{-\mathrm{kt}}\right)$$

where *C* represents the proportion of starch hydrolysed at time t (in minutes), *C*_∞_ denotes the equilibrium percentage of starch hydrolysed after 180 min, and k signifies the kinetic constant. The rate of starch digestion was quantified as the percentage of total starch released over time, while the hydrolysis index (HI) was calculated by dividing the area under the hydrolysis curve of each sample by the area of a reference sample (white bread) as reported by Goñi et al. (1997). The estimated Glycemic index (eGI) was calculated using the algorithm established by Granfeldt et al. (1992) shown below Equation (5).

(5) eGI=8.198+0.862HI

### 2.6 Statistical Analyses

All experiments were conducted in triplicate (*n* = 3) to ensure reproducibility and statistical robustness. The results are presented as mean ± standard deviation (SD). Statistical analyses were performed using SPSS version 26.0 (IBM Corp., Armonk, NY, USA), and one‐way analysis of variance (ANOVA) followed by Tukey's post hoc test was applied to determine significant differences among mean values (*p* < 0.05). Graphs were generated using OriginPro 2024b software (learning edition), and Microsoft Excel was used solely for preliminary data organization and visualization, not for inferential statistical testing.

## 3 Results and Discussion

### 3.1 Bread and Cookies Image Profile

**FIGURE 1:**
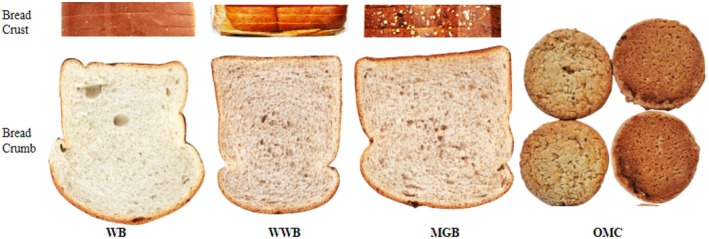
Photographs of bread & cookies samples. MGB, Multigrain Bread; OMC, Oatmeal Cookies; WB, White Bread; WWB, Whole Wheat Bread.

The crust and crumb grain characteristics of the three varieties of bread and oatmeal cookies were elucidated by the 2D image analysis. Figure 1 displays standard photos of bread crust and crumb. The visual examination of the bread crust indicates that both WB (White Bread) and MGB (Multigrain Bread) exhibit a comparable dark‐brown crust; however, the WWB (Whole Wheat Bread) has a lighter crust color. The Oatmeal cookies (OMC) exhibited a consistent exterior color on both the front and rear sides. The variance in crust color may be ascribed to differences in product composition and baking conditions, such as the extent of expansion during proving and the baking temperature (Gao et al. 2015).

The visual analysis of the crumb grain revealed that among the three bread kinds, WB exhibited a very porous crumb structure, whereas WWB and MGB had smaller, interconnected open holes.

### 3.2 Physicochemical Characteristics of Bread and Cookies

The moisture, fat, protein, fiber, total starch (TS), resistant starch (RS), and digestible starch (DS) content of the breads and cookies is summarized in Table 1. OMC had a lower moisture content (6.22 g/100 g) compared to WB (27.43 g/100 g), WWB (33.88 g/100 g), and MGB (32.26 g/100 g), although it possessed a superior starch concentration (40.40 g/100 g). Regarding digestibility, food items with elevated initial moisture content exhibited increased moisture levels at the swallowing point, as evidenced in studies on cookies, cakes, and white bread (Motoi et al. 2013), as well as among various bread types (Gao et al. 2019, 2015; Jourdren et al. 2016; Aleixandre *et al.,* 2019; Aleixandre et al. 2021). MGB exhibited the greatest protein content at 12.5 g, while WB and WWB demonstrated comparable protein levels at 9.33 g and 8.62 g per 100 g, respectively. OMC had the lowest protein content at 6.06 g per 100 g. The fiber content varies from 2.6 to 7.5, with WB exhibiting the lowest value and MGB the highest. The TS level in the bread and cookie samples was 45.49 g/100 g (white bread), 37.10 g/100 g (whole wheat bread), 33.65 g/100 g (multigrain bread), and 40.40 g/100 g (oatmeal cookie). The RS level differed among the samples, measuring 13.41 g/100 g for WB and 5.48 g/100 g for OMC.

**TABLE 1:** Physicochemical composition of the studied foods (g/100 g) “as is”.

Samples	Moisture (%)	Fat*	Protein*	Fiber*	TS	RS	DS (TS‐RS)
WB	27.43 ± 3.98^c^	6.67^b^	9.33^b^	2.6^d^	45.49 ± 3.2^a^	13.41 ± 1.20^a^	32.08 ± 1.2^b^
WWB	33.88 ± 1.33^a^	2.59^c^	8.62^c^	5.17^b^	37.10 ± 1.00^c^	8.71 ± 1.30^c^	28.39 ± 1.5^c^
MGB	32.26 ± 2.10^b^	3.75^b^	12.5^a^	7.5^a^	33.65 ± 0.98^d^	9.73 ± 2.50^b^	23.92 ± 2.0^d^
OMC	6.22 ± 0.25^d^	18.18^a^	6.06^d^	3.03^c^	40.40 ± 1.13^b^	5.48 ± 3.00^d^	34.92 ± 1.6^a^

*Note:* Values followed by different letters within a column are significantly different (*p* ≤ 0.05) (means a ≠ b).

Abbreviations: DS, Digestible Starch; MGB, Multigrain Bread; OMC, Oatmeal Cookies; RS, Resistant Starch; TS, Total Starch; WB, White Bread; WWB, Whole Wheat Bread.

* Values were not experimentally obtained but estimated from the nutritional facts tables supplied by the manufacturers (Table S1). Therefore, minor variations may exist between batches due to differences in raw materials and processing conditions.

### 3.3 Impact of Oral‐Proximal Gastric Digestion on Bolus Macrostructure Degradation and Dry Mass of Particulate Fraction

**FIGURE 2:**
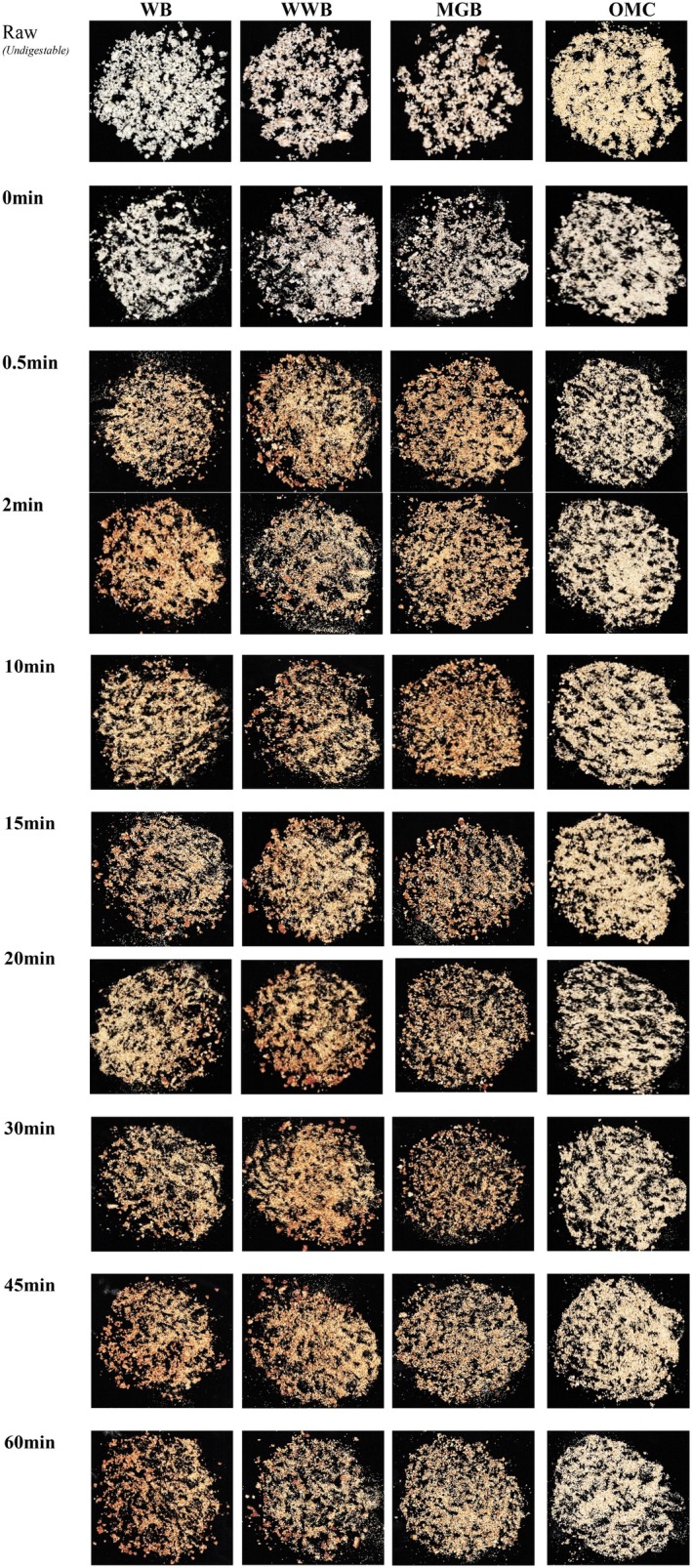
Visual representation of bolus macrostructure and dry particulate distribution during static oral‐proximal gastric digestion of wheat breads and oatmeal cookies. MGB, Multigrain Bread; OMC, Oatmeal Cookies; WB, White Bread; WWB, Whole Wheat Bread.

Figure 2 illustrates the particle morphology of the food bolus during oral–proximal gastric digestion for different bread and cookie samples. Clear differences were observed between samples digested with and without saliva addition, indicating the pivotal role of salivary α‐amylase in the early disintegration of starch‐based matrices. The lighter pellet color and reduced particle size in the saliva‐treated samples reflect enhanced enzymatic hydrolysis and matrix disruption compared with the undigested controls.

Quantitative analysis of dry mass distribution (Figure 2) demonstrated distinct degradation patterns among the tested products. The bread samples (WB and WWB) exhibited a gradual reduction in the coarse particle fraction (> 2 mm) throughout the 60‐min proximal gastric digestion, accompanied by a corresponding increase in fine particulate mass. This progressive transition suggests that starch gelatinization during baking produces a porous, hydrated structure that facilitates enzymatic penetration and mechanical fragmentation. The “zigzag” trend observed in the fine particle fraction may correspond to intermittent enzyme access as the bolus structure softens, followed by stabilization once the majority of accessible starch granules are hydrolysed. This behavior is consistent with previous observations for hydrated starch matrices and baked products (Freitas and Le Feunteun 2019).

In contrast, the oatmeal cookie (OMC) displayed a distinct degradation profile characterized by a dome‐shaped increase in fine particles during the first 30 min, followed by a plateau. This pattern likely reflects the denser, lipid‐rich matrix of cookies, where higher fat content and lower water activity limit enzyme diffusion and delay starch swelling. The initial phase of increased fine particle release may result from surface softening and partial disintegration of the outer cookie layer, while the plateau phase indicates resistance of the core structure to further enzymatic action. Such limited fragmentation and slower hydrolysis have been reported in compact baked goods with higher fat or sugar levels (Gao et al. 2019; Zhu et al. 2014). The continuous decline in coarse particle size in breads and the delayed response in cookies indicate that food matrix composition and processing strongly influence bolus disintegration kinetics. Products with higher porosity and moisture (bread) promote more effective mechanical and enzymatic breakdown, while compact, fat‐rich matrices (cookies) impede rapid enzyme access. These structural differences are likely to affect subsequent starch hydrolysis rates and postprandial glucose responses, as smaller bolus particle sizes have been associated with faster glucose release and higher Glycemic indices (Ranawana et al. 2010; Gao et al. 2019).

**FIGURE 3:**
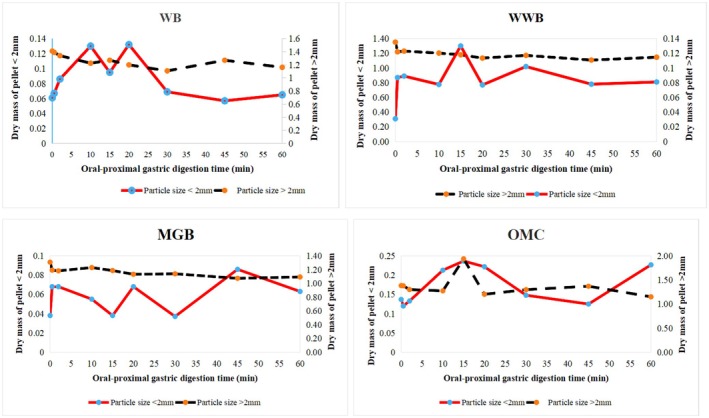
Changes in pellet particle size distribution during oral‐proximal gastric digestion of wheat bread, oatmeal cookies. Quantitative trends in fine and coarse particle fractions highlight differences in disintegration behavior among sample types. Bread matrices show a steady shift from coarse to fine particles, signifying efficient enzymatic and mechanical fragmentation, whereas the oatmeal cookie exhibits a delayed and limited reduction in coarse fraction due to its denser structure and lower water activity. These particle size dynamics suggest that food matrix composition governs the accessibility of starch to salivary α‐amylase, potentially influencing subsequent gastric hydrolysis and Glycemic response. MGB, Multigrain Bread; OMC, Oatmeal Cookies; WB, White Bread; WWB, Whole Wheat Bread.

Figure 3 summarizes these particle size trends, demonstrating that breads undergo more dynamic particle reduction than oatmeal cookies during oral–proximal gastric digestion. Overall, these findings highlight that the degree of bolus fragmentation and matrix softening during early digestion is closely related to food structure, composition, and salivary enzyme activity—all of which determine the accessibility of starch and phenolic compounds for subsequent gastric and intestinal hydrolysis.

### 3.4 Changes in Total Phenolic and Flavonoid Content During Oral‐Proximal Gastric Digestion

**FIGURE 4:**
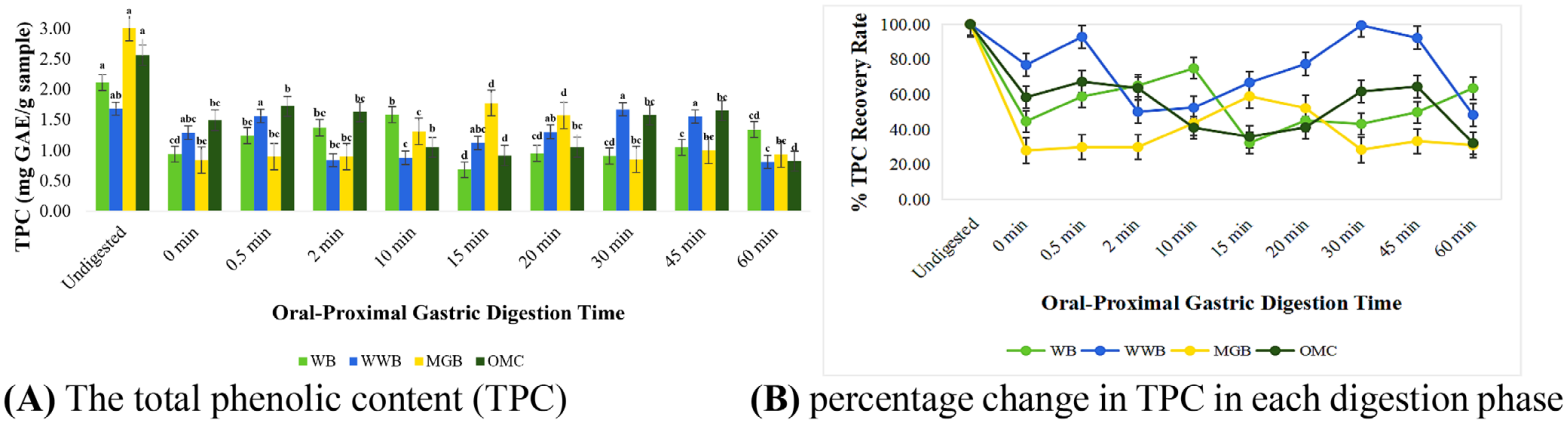
Changes in total phenolic content (TPC) & percentage recovery rate during oral–proximal gastric digestion of wheat breads and oatmeal cookie. Means sharing the same letter are not significantly different. Means with different letters are significantly different at *p* < 0.05. (A) The total phenolic content (TPC) (B) percentage change in TPC in each digestion phase. MGB, Multigrain Bread; OMC, Oatmeal Cookies; WB, White Bread; WWB, Whole Wheat Bread.

**FIGURE 5:**
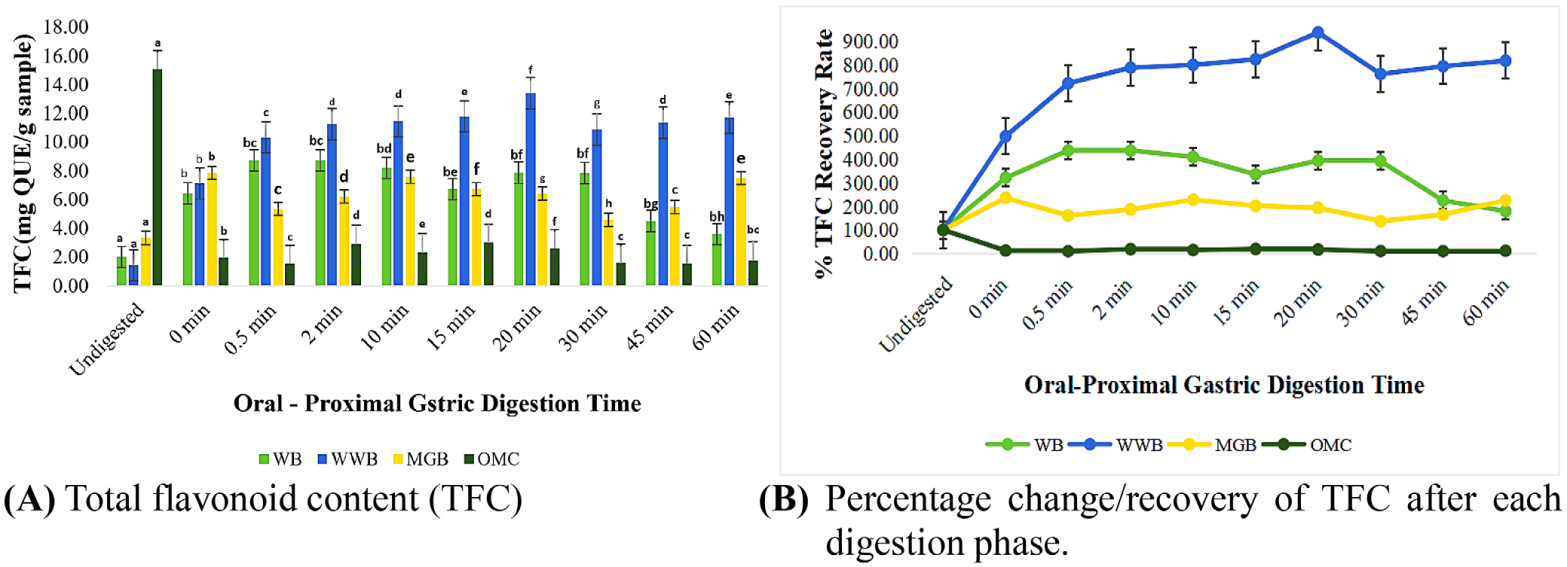
Changes in total flavonoid content (TFC) & percentage recovery rate during the oral–proximal gastric digestion phases of wheat breads and oatmeal cookies. Data are expressed as mean ± standard deviation (SD). Statistical differences among digestion phases within the same sample were evaluated using Welch's *t*‐test. Bars bearing different letters within the same sample indicate significant differences between digestion times (*p* < 0.05, ***p* < 0.001, ****p* < 0.0001), while bars sharing the same letter are not significantly different. (A) Total flavonoid content (TFC). (B) Percentage change/recovery of TFC after each digestion phase. MGB, Multigrain Bread; OMC, Oatmeal Cookies; WB, White Bread; WWB, Whole Wheat Bread.

The oral phase, including salivary α‐amylase activity, is often overlooked in studies examining the bioaccessibility of phenolic compounds (López‐Gámez et al. 2021a, 2021b; Carrasco‐Sandoval et al. 2021; Lyu et al. 2021; Eran Nagar et al. 2021; Mashitoa et al. 2021). Many authors assume that the short residence time and the limited action of salivary enzymes exert negligible influence on phenolic release, particularly in studies focusing on liquid or semi‐solid foods (Rasera et al. 2023). However, the updated INFOGEST protocol (Brodkorb et al. 2019) emphasizes inclusion of the oral phase in in vitro digestion models, as salivary interaction can alter the microstructural properties of starch‐based foods and influence the liberation of bound phytochemicals. The current study, therefore, examined how prolonged salivary incubation during the proximal gastric phase affects the release and bioaccessibility of total phenolic content (TPC) and total flavonoid content (TFC) in different bakery matrices (Figures 4 and 5).

As described in Section 3.3, the extent of bolus disintegration and particle size reduction plays a critical role in determining enzyme accessibility and diffusion within the food matrix. The porous and hydrated crumb structure of breads promotes salivary α‐amylase penetration, facilitating both starch hydrolysis and the release of phenolics and flavonoids that are weakly bound to polysaccharide or protein complexes. In contrast, the compact, fat‐rich matrix of cookies acts as a physical barrier to enzyme diffusion and solvent penetration, thereby restricting the release of these bioactive compounds during early digestion. During the oral–proximal gastric digestion phase (pH 6.5, 60 min), whole wheat bread (WWB) exhibited the highest TPC release, reaching approximately 99.4% of total phenolics at 30 min. This enhanced release can be attributed to the higher content of bound phenolic acids and flavonoids in whole grains, particularly in the bran and aleurone layers, as well as the greater porosity of the whole wheat bread matrix, which facilitates water absorption and enzymatic access. In contrast, the multigrain bread (MGB) showed the lowest phenolic release (28.35%), likely due to its denser crumb structure and the presence of mixed cereal components, where cross‐linking between polyphenols, proteins, and dietary fibers may limit solubility and enzymatic liberation (Zheng et al. 2018).

Flavonoid recovery followed a similar trend but with notable quantitative differences among products. Whole wheat bread again displayed the highest TFC recovery (approximately 938.06% at 20 min), suggesting a potential transformation or release of bound flavonoids during matrix breakdown. This unusually high recovery could be explained by the disruption of hydrogen bonding and hydrophobic interactions between flavonoids and macromolecules (proteins, starch, or cell wall polysaccharides) during enzymatic hydrolysis and mechanical disintegration. In contrast, both MGB and the oatmeal cookie (OMC) exhibited lower TFC and TPC recoveries, likely reflecting the stronger encapsulation of phenolics within lipid–protein matrices and lower water activity in cookies, which hinder diffusion and solubilization. The pronounced difference between cookies and breads thus reflects their fundamentally different structural and compositional characteristics. Breads, particularly whole wheat bread, possess open, hydrated networks that favor enzyme diffusion and phenolic release, while cookies contain compact, hydrophobic matrices where phenolics remain bound or entrapped. Additionally, the higher fat and sugar content of cookies may form complexes with flavonoids, reducing their extractability during digestion. These structural–chemical interactions explain the significant variability in recovery rates observed between the product types. Overall, these findings highlight that food microstructure, moisture content, and matrix composition are key determinants of phenolic and flavonoid bioaccessibility during early digestion. The oral–proximal gastric phase, often overlooked, plays a critical role in initiating these release processes, thereby influencing the total antioxidant potential available for intestinal absorption and subsequent metabolism. Therefore, the polarities, molecular weights, structural variations, and concentrations of approximately eight thousand phytochemicals present in plants can influence the digestive process in various ways, resulting in differing bioavailability within the body (Schulz et al. 2017; Aylanc et al. 2021).

### 3.5 Phenolic and Flavonoid Bioaccessibility Level After Each In Vitro Digestion Phase

**FIGURE 6:**
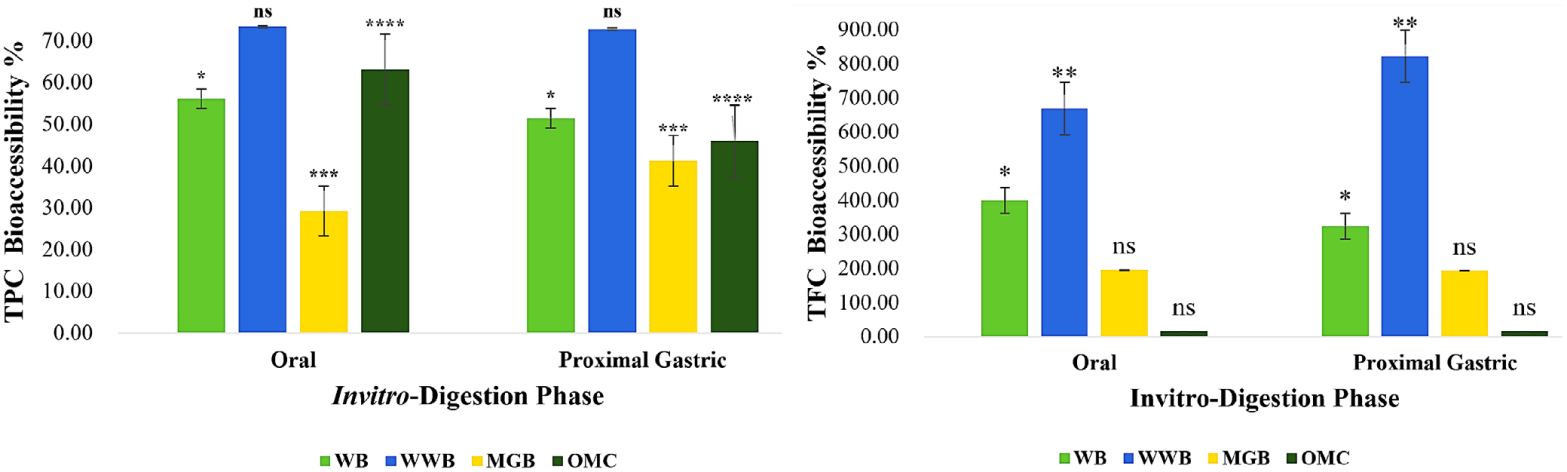
Percentage bioaccessibility of total phenolic content (TPC) and total flavonoid content (TFC) during oral and proximal gastric digestion of wheat breads and oatmeal cookies. Data are presented as mean ± SD. Statistical significance between the oral and proximal gastric phases was determined using Welch's t‐test. Asterisks indicate significant differences between corresponding bars (*p < 0.05, ***p < 0.001, ****p < 0.0001). MGB, Multigrain Bread; OMC, Oatmeal Cookies; WB, White Bread; WWB, Whole Wheat Bread; ns, not significant.

The bioaccessibility of total phenolic content (TPC) and total flavonoid content (TFC) exhibited distinct and irregular trends across the oral and proximal gastric digestion phases (Figures 4B, 5A,B and 6). Bioaccessibility represents the proportion of ingested polyphenols that become available for intestinal absorption after digestion, ultimately influencing their bioavailability and physiological effects (Luzardo‐Ocampo et al. 2017; Pinto, Moreira, Švarc‐Gajić, et al. 2023). In the present study, the highest average recovery rates of 73.21% for TPC and 822.68% for TFC were observed, demonstrating considerable variation among food matrices. The unusually high flavonoid bioaccessibility (> 800%) likely results from multiple overlapping factors. First, enzymatic hydrolysis and the alkaline‐to‐acidic pH transition during the proximal gastric phase may liberate bound flavonoids and promote the biotransformation of complex glycosides into simpler aglycones and phenolic derivatives such as phenylvalerolactones and phenylvaleric acids (Shang et al. 2024; Quan et al. 2020). These transformation products can react with Folin–Ciocalteu and aluminum chloride reagents, leading to apparent overestimation in colorimetric assays (Ng and See 2019; Li et al. 2022). Second, disruption of the protein–polyphenol and starch–polyphenol complexes during digestion increases the solubilization of previously bound flavonoids, thereby inflating apparent recovery values. Lastly, analytical limitations should also be considered, as the spectrophotometric methods used primarily quantify soluble phenolics while underestimating insoluble polymeric forms (Pinto, Moreira, Vieira, et al. 2023).

Matrix composition and microstructure critically influenced the extent of phenolic and flavonoid release. Whole‐wheat bread (WWB) exhibited the highest TPC and TFC bioaccessibility, attributable to its porous, hydrated structure and the presence of bran‐associated phenolics that become accessible after salivary α‐amylase action. In contrast, multigrain bread (MGB) and oatmeal cookie (OMC) showed markedly lower recoveries, reflecting the restricted enzyme diffusion within their compact, lipid‐rich matrices and the presence of multiple grain types, which may result in complex phenolic–fiber interactions that hinder solubilization. These observations are consistent with previous findings that matrix porosity, particle size, and hydration significantly affect enzyme accessibility and phenolic release during digestion (Freitas and Le Feunteun 2019; Gao et al. 2021; Panagopoulou et al. 2021).

Interestingly, while most studies report a gradual decline in TPC and TFC across the digestive process (Schulz et al. 2017; Ng and See 2019), our results revealed partial increases during the oral and proximal gastric phases, particularly in WWB. Such discrepancies may be explained by differences in substrate type, phenolic composition, and digestion simulation conditions. For instance, polyphenol‐rich foods such as Moringa leaves (Dou et al. 2019), bee pollen (Aylanc et al. 2021), and hawthorn (Zheng et al. 2018) also show enhanced release during early digestion due to rapid enzymatic exposure and mechanical breakdown. The extended salivary interaction period adopted in this study may therefore have promoted a higher degree of early phenolic and flavonoid liberation compared to conventional short‐term oral simulations.

Overall, these findings underscore that polyphenol bioaccessibility is governed by the interplay between food matrix characteristics (porosity, lipid and fiber content), enzymatic activity, and digestion pH that facilitate their degradation and biotransformation into simpler compounds (Pinto, Moreira, Vieira, et al. 2023). The exceptionally high apparent recovery of flavonoids does not necessarily indicate superior bioavailability but rather reflects the combined effects of matrix disintegration, compound transformation, and analytical overestimation. The TPC and TFC contents of digested fractions may be underestimated because the methodologies used only quantify soluble phenolic compounds, overlooking condensed phenolic compounds, proanthocyanidins, and other insoluble polymeric phenolic compounds that are not extracted in the aqueous digestion media. Furthermore, soluble and insoluble fiber prevalent in plant food matrices may disrupt spectrophotometric tests for quantifying TPC and TFC contents (Pinto, Moreira, Vieira, et al. 2023). Future studies integrating targeted metabolite profiling (e.g., LC–MS/MS) would be valuable to confirm the identity of transformation products and provide a more accurate quantification of bioaccessible phenolics and flavonoids.

### 3.6 Release of Reducing Sugar During In Vitro Oral‐Proximal Gastric Digestion of Bread and Cookies Bolus

**FIGURE 7:**
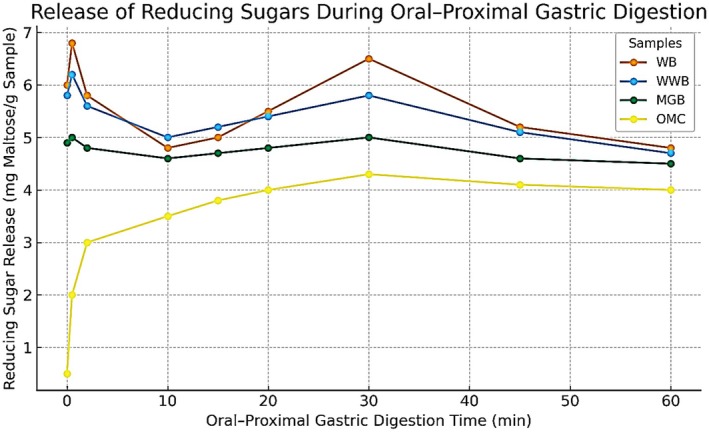
*Release of reducing sugars (expressed as mg maltose/g sample) during* in vitro *oral–proximal gastric digestion of wheat‐based breads and oatmeal cookies*. Data are presented as mean ± SD (*n* = 3). The line chart illustrates the time‐dependent release dynamics of reducing sugars from white bread (WB), whole wheat bread (WWB), multigrain bread (MGB), and oatmeal cookie (OMC).

The dynamic release of reducing sugars during the oral–proximal gastric digestion of the tested wheat bakery products is presented in Figure 7. The line profiles reveal time‐dependent variations in reducing sugar release, displaying distinct patterns among the different food matrices (WB, WWB, MGB, and OMC). Overall, the release trends confirm that the rate and extent of maltose liberation are strongly influenced by the structural and compositional characteristics of each matrix, reflecting the complex interplay between enzymatic hydrolysis, substrate accessibility, and food matrix characteristics. White bread (WB) exhibited the highest initial release of reducing sugars, reaching a peak of approximately 6.8 mg maltose/g at 0.5 min, followed by a transient decline and a secondary rise around 30 min. This dual‐peak behavior may reflect the combined effects of surface starch hydrolysis during early mastication and subsequent exposure of entrapped starch as the bolus undergoes softening and hydration (Sharma et al. 2020). This trend also suggests that the highly porous and hydrated crumb structure of WB allows rapid enzyme penetration and efficient starch hydrolysis by salivary α‐amylase during the early stages of digestion. In contrast, whole wheat bread (WWB) showed a more gradual and consistent profile, suggesting a more cohesive matrix that moderates enzymatic accessibility despite comparable starch content, likely due to the presence of bran particles and higher fiber content that restrict water diffusion and enzyme accessibility, moderating the rate of starch breakdown.

For multigrain bread (MGB), reducing sugar levels fluctuated mildly, indicating the buffering effect of mixed grain components (e.g., oat, rye, millet) and dietary fiber, which can hinder α‐amylase diffusion and limit substrate—enzyme interactions. Meanwhile, oatmeal cookies (OMC) initially exhibited the lowest sugar release (≤ 2 mg maltose/g) but showed a steady increase over time, approaching 4 mg maltose/g by 30 min. This delayed release pattern reflects the dense, lipid‐rich structure and higher thermal processing degree of cookies, which can restrict enzymatic penetration early on but gradually yield sugars as the matrix softens and swells during digestion (Elechi 2024). Furthermore, the presence of fats and Maillard reaction products in baked cookies may initially inhibit α‐amylase activity by forming starch—lipid—protein complexes; however, progressive hydration and matrix softening during digestion gradually enhance enzyme access, resulting in a late‐phase rise in reducing sugar release (Sharma et al. 2020; Gao et al. 2021). These contrasting release behaviors highlight that matrix integrity, processing intensity, and macronutrient interactions collectively dictate sugar release kinetics. Overall, the variable reducing sugar release profiles observed across the samples emphasize that starch digestibility is governed by matrix porosity, hydration capacity, and compositional factors such as lipid and fiber content. The liquefying action of salivary α‐amylase during oral processing accelerates maltose generation, while the transitional pH conditions in the proximal gastric phase sustain moderate enzymatic activity before inactivation at lower pH levels. The line chart presentation clarifies these temporal relationships, demonstrating how product‐specific structures influence the enzymatic breakdown of starch during oral‐proximal gastric digestion.

### 3.7 Glucose Release and Hydrolysed Starch Content During In Vitro Oral‐Proximal Gastric Digestion of Bread and Cookies Bolus

**FIGURE 8:**
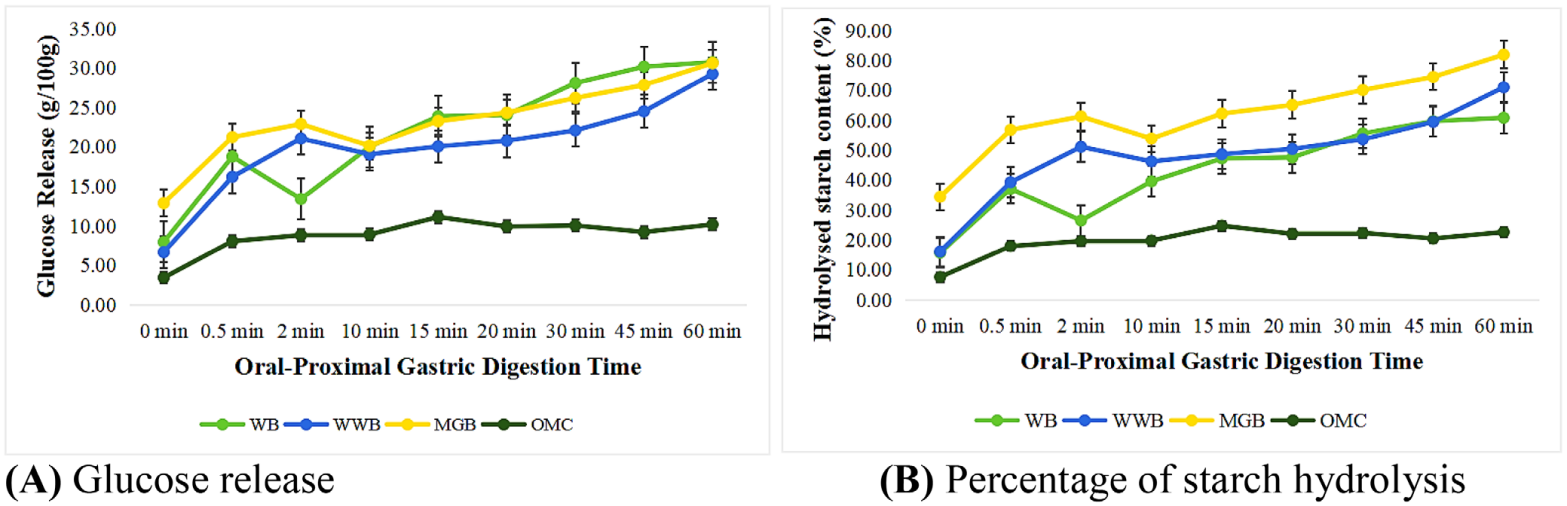
Glucose release and changes in percentage of starch hydrolysis during the simulated oral‐proximal gastric digestion process. (A) Glucose release. (B) Percentage of starch hydrolysis.

Figure 8 illustrates the glucose release and variations in the proportion of starch hydrolysed during the simulated oral‐proximal stomach digesting process. The amount of sugar released escalated swiftly in a nonlinear fashion during the duration of 60 min (8A). All bread samples, regardless of beginning starch content (Table 1) and component compositions (Table S1), had comparable glucose levels after 60 min. The particle size of the flours utilized in bread baking may contribute to this, since whole grain flour is produced by grinding the entire grain kernel, which might enhance its vulnerability to hydrolysis. The incorporation of coarse or whole grains (e.g., rolled oats in OMC) yields a reduced glucose and Glycemic index value relative to the use of finely milled whole grain flour (e.g., wholemeal bread). Consequently, in this investigation, the increased vulnerability of the wheat bread samples to salivary action may be attributed to a greater quantity of tiny particles, which are more readily degraded by α‐amylase (Gamero et al. 2019). The particle size of milled cereal grains significantly impacts both in vitro and in vivo starch digestibility, hence affecting the postprandial Glycemic response (PPGR) among many dietary component and matrix parameters (Ranawana et al. 2011). As particle size diminishes, the surface area accessible to α‐amylase expands, resulting in an accelerated rate of starch digestion (Ranawana et al. 2010). Moreover, coarse milling of grain into particles exceeding the size of individual endosperm cells (100–150 μm) ensures they maintain a significant level of integrity (Mulargia et al. 2025). The cell walls of the endosperm serve as physical barriers, obstructing the passage of α‐amylase to the encapsulated starch granules. The larger particle size of milled wheat results in a reduction of the in vitro starch digestion rate and the in vivo postprandial glycemic response (Edwards et al. 2015; Korompokis et al. 2019).

Consequently, the oatmeal cookies (OMC) demonstrated a linear glucose release profile at lower values in contrast to the other breads, although possessing a high initial starch content (40 g/100 g). This contradicts the findings of Jaime‐Fonseca et al. (2016), who observed that the increase in glucose concentration was more pronounced with increased starch content due to elevated luminal glucose levels. The reduced glucose release in OMC may be ascribed to product characteristics, specifically the input ingredient of rolled oats (S1), which aligns with the findings of Gamero et al. (2019), who noted that cookies made with oat flour released more glucose than those made with larger flakes, as the oat‐flour cookie matrix is more prone to enzymatic hydrolysis.

Figure 8B illustrates the alterations in starch hydrolysis (%), indicating that the various bread samples (WB, WWB, and MGB) demonstrated a comparable trend in hydrolysed starch content, as evidenced by glucose release. By the conclusion of the oral phase (0–2 min), a minimum of 26.45% (WB), 35.53% (WWB), and 50.84% (MGB) of bread starch had undergone hydrolysis, with 60.84% (WB), 71.01% (WWB), and 81.91% (MGB) of starch hydrolysed by the end of the 60‐min proximal gastric digestion. Furthermore, a minimum of 19% of cookie starch (OMC) had undergone hydrolysis at the conclusion of the oral phase, with an additional 22% hydrolysed within the initial 30 min and by the completion of proximal stomach digestion. The partly hydrolysed starch content documented in this study, albeit elevated, aligns with the trend identified by Freitas et al. (2018). Variations in results may arise from sample parameters, saliva volume, and digesting protocols (static versus dynamic, saliva alone versus saliva combined with pepsin). Our research aligns with the findings of Nadia et al. (2022), who observed that hydrolysed starch quantity increased with an extended proximal phase across all meal types (couscous, rice couscous, pasta, rice noodle, and rice grain), irrespective of the distal phase duration. Previous investigations have also identified comparable glucose release and enhanced hydrolysed starch content in chewed rice (Ranawana et al. 2010; Tamura et al. 2017).

Woolnough et al. (2010) examined starch hydrolysis during the oral phase and discovered that 13% of bread starch was hydrolysed (either entirely to glucose or partially to dextrins) by HSA. They concluded that the presence of this enzyme did not affect the starch digestion profiles during in vitro intestinal digestions, thereby questioning its significance in the digestive process (Freitas et al. 2018). However, their findings resulted from employing a strategy that mimicked an instantaneously acidified stomach phase, causing quick suppression of amylolytic activity following the oral phase, which does not accurately reflect human biological reality (Freitas et al. 2018; Nadia et al. 2022). Freitas et al. (2018) and Freitas and Le Feunteun (2019) noted that the most rapid starch hydrolysis occurred in bread, with up to 80% of starch released within the initial 20 min of gastric digestion, indicating that HSA may facilitate significant amylolysis prior to chyme entering the small intestine. Furthermore, they demonstrate that starch hydrolysis was more significant during the gastric phase, when about 60% of starch was converted into oligosaccharides, compared to intestinal digestion, which resulted in an additional rise of around 20%. Consequently, our results reinforce the conclusions of Freitas et al. (2018), Freitas and Le Feunteun (2019) and Nadia et al. (2022), further affirming the biological importance of the oral‐proximal stomach phase in food digestion research. The residual starches that were not hydrolysed indicated the presence of starch resistance (Table 1) to oral‐gastric digestion, likely attributable to the sequential actions of salivary amylase and pepsin in vivo, as well as the entrapment of starch within the gluten matrix of bread (Freitas et al. 2018).

### 3.8 Kinetics of Starch Hydrolysis and Predicted Glycaemic Index (GI)

**FIGURE 9:**
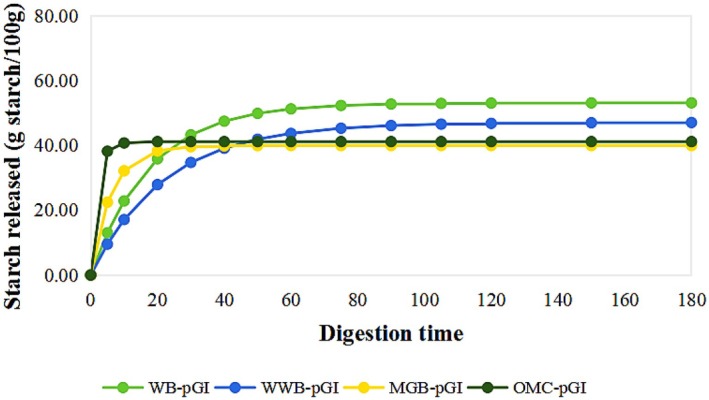
Kinetics of starch hydrolysis and predicted Glycemic index (eGI) of wheat bread and cookie samples during in vitro oral‐proximal gastric digestion. MGB, multigrain bread; OMC, Oatmeal Cookies; pGI, predicted glycemic index; WB, white bread; WWB, whole wheat bread.

The kinetics of starch hydrolysis and the corresponding predicted Glycemic index (GI) of the wheat breads and oatmeal cookies are presented in Figure 9, with the main parameters summarized in Table 2. Starch digestion kinetics provide critical insight into postprandial Glycemic responses, as the rate of glucose liberation governs intestinal absorption and subsequent metabolic impact (Jenkins et al. 1982). Across all tested products, rapidly digestible starch (RDS) constituted the major starch fraction, consistent with the high degree of starch gelatinization and surface accessibility typically observed in baked wheat products. However, the proportion of slowly digestible starch (SDS) varied significantly among samples, revealing key differences in matrix structure and compositional integrity.

**TABLE 2:** Kinetic parameters of starch hydrolysis and predicted Glycemic index (eGI) of wheat breads and oatmeal cookies.

Samples	RDS (g/100 g)	SDS (g/100 g)	C∞	K (min^−1^)	AUC	HI	eGI
WB	35.8 ± 0.06^b^	17.2 ± 0.27^a^	5.31 ± 0.21^a^	0.056 ± 0.006^d^	859 ± 24^a^	100 ± 2^a^	94 ± 10^a^
WWB	27.9 ± 0.24^c^	18.9 ± 0.46^a^	4.71 ± 0.25^b^	0.046 ± 0.010^c^	739 ± 15^b^	86 ± 2^b^	82 ± 10^b^
MGB	38.3 ± 0.06^ab^	1.6 ± 0.13^b^	3.99 ± 0.19^c^	0.168 ± 0.04^b^	692 ± 27^d^	81 ± 3^c^	78 ± 11^c^
OMC	41.1 ± 0.13^a^	0.00 ± 0.00^b^	4.12 ± 0.13^c^	1.704 ± 1.86^a^	729 ± 20^c^	85 ± 2^b^	81 ± 10^b^

*Note:* Values represent mean ± SD (*n* = 3). Values followed by different letters within a column are significantly different (*p* ≤ 0.05). MGB displayed a markedly higher SDS content and lower eGI than WB and WWB, reflecting restricted enzymatic accessibility caused by coarse‐grain particle integrity and high fiber content. Conversely, OMC exhibited negligible SDS yet maintained a lower eGI, attributable to lipid–starch complex formation and matrix compactness that limit enzyme diffusion. Statistical significance (*p* < 0.05) indicates distinct digestibility behaviors among sample types.

Abbreviations: AUC, area under the hydrolysis curve after 180 min; C_∞_, equilibrium concentration; eGI, estimated glycemic index; HI, hydrolysis index; K, kinetic constant; MGB, multigrain Bread; OMC, oatmeal cookies; RDS, Rapid digestible starch; SDS, Slowly digestible starch; WB, White Bread; WWB, Whole Wheat Bread.

The multigrain bread (MGB) exhibited a significantly higher SDS fraction compared with other breads, despite an overall moderate hydrolysis rate. This may be attributed to the heterogeneous grain composition, where intact or partially disrupted grains and high‐fiber components act as physical barriers to enzymatic diffusion (Edwards et al. 2015; Johansson et al. 2018). The presence of cell wall polysaccharides, β‐glucans, and resistant starch granules within the coarse‐grain matrix likely retards starch gelatinization and amylolysis. Consequently, MGB demonstrated a lower predicted Glycemic index (GI = 78 ± 11) compared to WB and WWB, aligning with previous findings that multigrain and intact‐particle breads exhibit attenuated Glycemic potency (Akila et al. 2019; Freitas, Souchon, and Le Feunteun 2022).

Conversely, the oatmeal cookie (OMC) displayed an extremely low SDS content (0.00 g/100 g) but also a relatively low estimated GI compared to white bread. This seemingly paradoxical observation can be rationalized by considering the combined effects of matrix compactness, lipid–starch interactions, and macronutrient composition. OMC has a dense, hydrophobic structure due to high fat and sugar content, which restricts water penetration and enzyme diffusion during early digestion (Gamero et al. 2019). Although its SDS fraction is minimal, the rate of glucose release is slowed by limited enzyme accessibility, explaining its lower eGI. Furthermore, the presence of amylose–lipid complexes formed during baking can further reduce the hydrolysis rate of gelatinized starch, mimicking SDS‐like behavior despite its negligible analytical detection (Mishra et al. 2012). These findings underscore that SDS content alone does not fully predict Glycemic behavior—structural and compositional attributes of the food matrix play an equally important role. The apparent discrepancies between OMC's low SDS and low eGI are therefore attributable to the combined inhibitory effects of fat entrapment, reduced porosity, and starch–lipid complexation, rather than the intrinsic starch fraction alone (Berg et al. 2012; Edwards et al. 2015).

Statistical analysis confirmed significant (*p* < 0.05) variation in the kinetic constant (*k*) and equilibrium concentration (*C∞*) across samples, reflecting their diverse microstructures and processing histories. The area under the hydrolysis curve (AUC_180_) for MGB and OMC was notably lower than for WB, consistent with their reduced enzymatic susceptibility and corresponding lower Glycemic response potential. Collectively, these results highlight that both grain particle integrity (as in MGB) and matrix compaction or lipid encapsulation (as in OMC) can attenuate starch digestibility and Glycemic response—albeit through distinct mechanisms. This mechanistic understanding reinforces the importance of food microstructure design in modulating carbohydrate quality and metabolic outcomes.

The kinetic digestion rate of starch was evaluated to determine both the extent and rate of starch hydrolysis during in vitro digestion (Figure 9). The curves reveal characteristic first‐order kinetic behavior, with a rapid initial phase of starch release followed by a gradual plateau, indicating enzyme saturation and substrate depletion over time. Among the samples, white bread (WB) exhibited the highest and fastest starch hydrolysis, reaching equilibrium (C∞ ≈ 65–70 g starch/100 g) within 60 min. This rapid hydrolysis reflects its highly porous crumb structure, low fiber content, and uniform starch–protein matrix, which together facilitate extensive enzyme accessibility and diffusion. In contrast, whole wheat bread (WWB) and multigrain bread (MGB) demonstrated slower hydrolysis rates and lower equilibrium concentrations. The reduced digestibility of WWB and MGB can be attributed to the physical barrier effects of bran layers, the presence of intact grain particles, and higher fiber fractions that restrict α‐amylase diffusion into the starch granule core. Specifically, MGB maintained the lowest starch hydrolysis plateau (~40 g starch/100 g), consistent with its dense structure and abundant insoluble fiber. These findings support previous observations that coarse‐grain inclusion and unruptured cell walls markedly impede starch bioaccessibility (Berg et al. 2012; Akila et al. 2019).

Oatmeal cookies (OMC) presented a unique digestion pattern: despite showing a moderate hydrolysis rate similar to MGB, OMC reached a slightly higher final starch release. This behavior may stem from the compact, fat‐rich matrix of oat‐based systems, in which lipid–starch complex formation and limited porosity slow enzymatic penetration. Additionally, the finer particle size of oat flour—produced by complete groat milling—creates a microstructure more susceptible to initial salivary hydrolysis (Gamero et al. 2019). However, the presence of β‐glucans and bound lipids subsequently reduces the overall digestibility and Glycemic index, resulting in a paradoxical combination of rapid early hydrolysis but low eGI.

Collectively, these findings confirm that starch digestion kinetics are governed not solely by carbohydrate type but by food microstructure and matrix integrity. While WB exhibited the most rapid hydrolysis and highest predicted GI, WWB and MGB displayed moderate‐to‐low rates due to structural resistance from wholemeal particles. OMC's digestion profile underscores the importance of lipid‐starch and fiber‐starch interactions in modulating enzymatic accessibility. These interactions explain why foods with similar starch contents can exhibit markedly different Glycemic responses, reinforcing the role of processing and matrix design in controlling Glycemic potency.

### 3.9 Comparative Analysis of the Predictive GI and the Stimulated Oral‐Proximal Gastric *Invitro* Digestion

**FIGURE 10:**
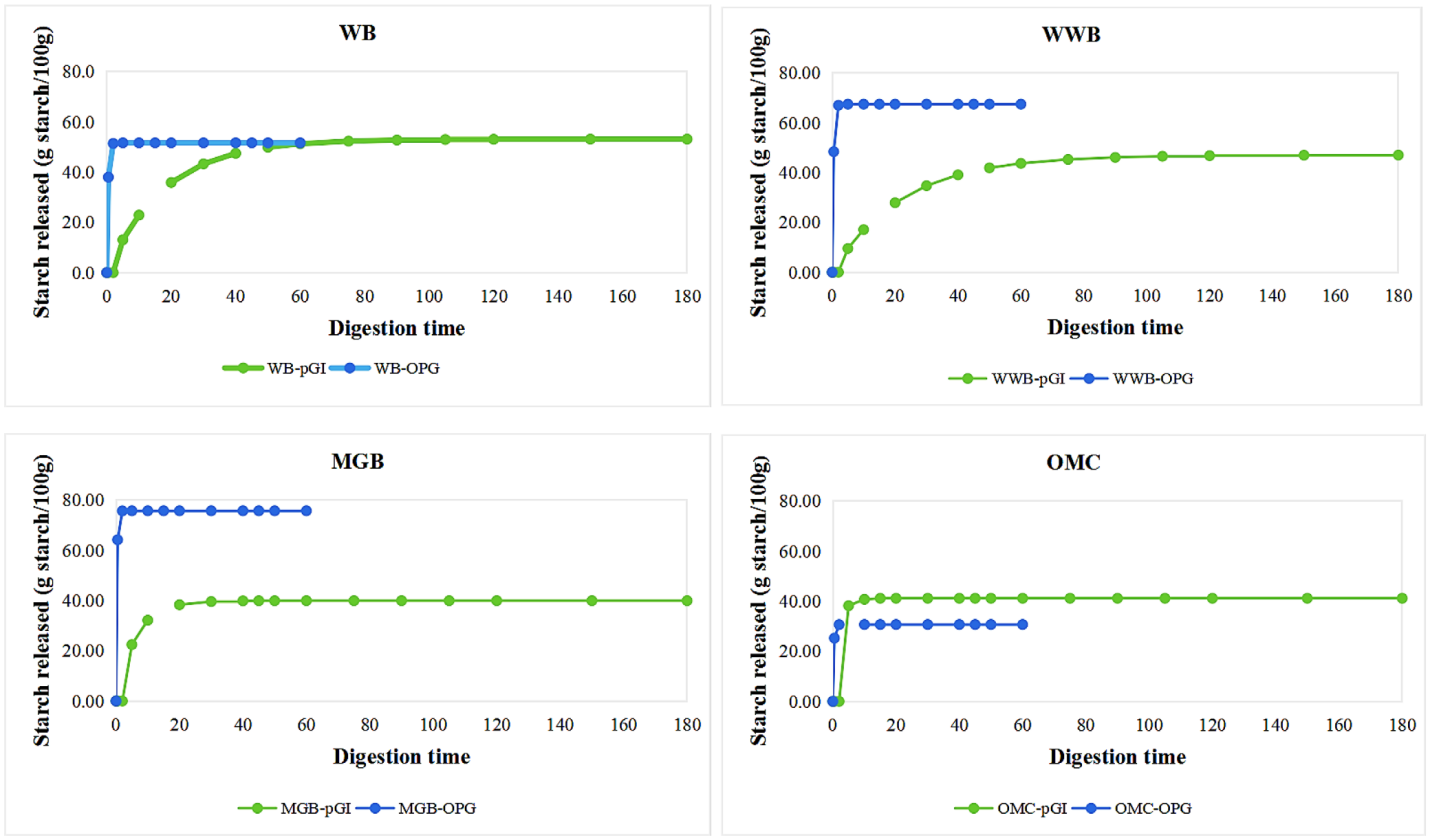
Kinetics of starch hydrolysis: predicted glycemic index versus oral‐proximal gastric in vitro digestion MGB, multigrain bread; OMC, oatmeal cookies; pGI, predicted glycemic index; OPG, oral‐proximal gastric digestion; WB, white bread; WWB, whole wheat bread.

Figure 10 presents a comparison of starch release kinetics obtained using the in vitro methodology for Glycemic index (GI) prediction (Goñi et al. 1997) and our stimulated oral‐proximal stomach digestion model. Despite differences in enzyme composition and experimental conditions, starch release during the oral‐proximal stomach phase was highly similar for WB and OMC. In contrast, WWB and MGB showed significantly higher starch release but lower predicted GI compared to WB, consistent with prior observations on wheat and gluten‐free pastas (Freitas and Le Feunteun 2019). These results suggest that the structural characteristics of bread, rather than solely its macronutrient composition, play a central role in modulating starch hydrolysis. For example, breads with porous textures and highly gelatinised starch, typical of certain wholemeal breads, may allow rapid enzymatic access, whereas bran fractions can disrupt gluten networks, modulating digestion kinetics (Fardet et al. 2006; Freitas, Souchon, and Le Feunteun 2022). This aligns with findings that macro‐ and microstructural properties can influence GI outcomes more than composition alone (Freitas, Souchon, and Le Feunteun 2022).

Although this study did not experimentally manipulate mastication, previous research suggests that the extent of oral processing can influence early starch hydrolysis by salivary α‐amylase, potentially affecting subsequent Glycemic responses (Ranawana et al. 2010; Freitas, Souchon, and Le Feunteun 2022). Our results support this mechanistic framework: breads requiring greater bolus formation due to high hydration or crust hardness may allow enhanced saliva integration, facilitating early starch breakdown and contributing to interindividual variations in GI. Furthermore, our study highlights the contribution of early‐phase starch hydrolysis in the oral‐proximal gastric environment. Starch release during this phase was substantially elevated for breads with extended hydration capacity or softer structures, consistent with enhanced enzymatic accessibility. These findings support the notion that multiple factors converge to influence GI, particularly the decreased hydrolysis of starch by human salivary α‐amylase (HSA) during oro‐gastric digestion (Freitas, Souchon, and Le Feunteun 2022). Research conducted by various groups indicates an elevated Glycemic response associated with an extended oral phase (Freitas, Souchon, and Le Feunteun 2022; Freitas, Boué, et al. 2022; Nadia et al. 2022). Overall, the comparative analysis demonstrates that the stimulated oral‐proximal gastric model closely mirrors predictive GI estimates, while also providing additional insight into structural and enzymatic determinants of starch digestion. This underscores the multifactorial nature of starch digestion and supports the use of mechanistic in vitro models to complement GI prediction approaches.

## 4 Conclusions

This study underscores the pivotal but often overlooked role of the oral and proximal gastric phases in shaping the digestibility and metabolic fate of starch‐based foods. During these early digestive stages, the sustained activity of salivary α‐amylase (HSA) and the mechanical forces of mastication and bolus formation governed particle disintegration, markedly influencing subsequent starch hydrolysis kinetics. The observed shifts in particle size distribution—particularly the generation of fine particles (< 2 mm)—demonstrate that matrix disruption is a key determinant of nutrient bioaccessibility and enzymatic accessibility. The irregular release patterns of reducing sugars and phenolic compounds across food matrices highlight that starch digestibility is highly dependent on structural and compositional factors. Whole and multigrain breads exhibited slower starch hydrolysis and reduced Glycemic potency due to the encapsulation of starch within intact grain fragments and higher fiber content, which limited enzyme diffusion. In contrast, oatmeal cookies, despite their low slowly digestible starch (SDS) content, showed a lower estimated Glycemic index (eGI), likely driven by lipid‐starch and β‐glucan interactions that hindered enzyme accessibility during digestion. These findings emphasize that the physicochemical microstructure of the food matrix—not starch content alone—governs the rate and extent of starch digestion and, consequently, the Glycemic response.

Mechanistically, the study provides evidence that the prolonged oral–proximal gastric exposure phase modulates the interplay between mechanical disintegration and α‐amylase‐mediated hydrolysis, establishing early determinants of postprandial glycaemia. The positive correlation between starch digestion kinetics during these stages and the estimated Glycemic index supports the predictive validity of early‐phase digestion models for in vivo metabolic outcomes. This mechanistic understanding offers new insights into designing cereal‐based products that strategically modulate salivary α‐amylase activity to manage Glycemic impact without compromising sensory quality.

### 4.1 Limitations and Future Directions

This study included one commercially available oatmeal cookies selected as a structural and compositional contrast to wheat breads. It was not intended to represent the full diversity of oat‐based bakery formulations available on the market. In contrast, three bread types were examined to capture variability associated with grain composition and product labeling categories. Because detailed industrial processing parameters were not available and no independent structural or thermal characterization was conducted, interpretations regarding differences in starch gelatinisation and matrix architecture are inferred from formulation and product classification rather than directly measured processing variables. Future research should incorporate a broader range of oat‐based products and include structural characterization techniques (e.g., DSC, microscopy, or X‐ray diffraction) to strengthen mechanistic understanding of how processing influences oral‐proximal digestion, starch hydrolysis, and phenolic bioaccessibility.

While the in vitro model used effectively simulates oral and proximal gastric digestion, it cannot fully replicate the complexity of in vivo conditions, including hormonal regulation, gastric motility, and interindividual variations in saliva composition and chewing behavior. Future research should incorporate in vivo validation and consider variations in oral physiology, enzyme activity, and food matrix interactions to refine predictive accuracy. Additionally, exploring food formulation strategies that integrate enzyme inhibitors, fiber networks, or resistant starch structures may help develop starch‐based products with improved metabolic health outcomes.

It is important to acknowledge that the quantification of total flavonoid content (TFC) in this study was based on colorimetric assays, which, although widely used for comparative analysis, may be susceptible to matrix interferences and variations in reaction specificity. The observed discrepancy between the release patterns of total phenolic content (TPC) and TFC may partly stem from the differential reactivity of non‐flavonoid phenolics or the influence of other co‐extracted compounds that can interfere with aluminum chloride complex formation. Consequently, the colorimetric method may not fully capture the compositional diversity or individual flavonoid dynamics within the digesta. Future studies employing chromatographic techniques such as high‐performance liquid chromatography (HPLC) or LC–MS would provide a more precise quantification and characterization of specific flavonoid subclasses, enabling a clearer interpretation of their bioaccessibility and digestive stability.

## Author Contributions

**Jasper Okoro Godwin Elechi:** conceptualization, investigation, methodology, validation, writing – review and editing, visualization, writing – original draft, formal analysis. **Roberto Cannataro:** investigation, formal analysis, supervision, writing – review and editing, writing – original draft. **Erika Cione:** conceptualization, investigation, writing – original draft, writing – review and editing, visualization, methodology, supervision. **Diana Marisol Abrego‐Guandique:** writing – original draft, writing – review and editing, formal analysis, supervision. **Nicola Gasparre:** conceptualization, investigation, writing – original draft, methodology, writing – review and editing, formal analysis, supervision.

## Ethics Statement

This study did not involve clinical intervention, patient recruitment, or human subject experimentation. Saliva samples were obtained from healthy adult volunteers through a one‐time, non‐invasive donation for use in in vitro digestion experiments conducted at the University of Calabria, Italy. All sample collection, laboratory analyses, and experimental procedures were performed in Italy. The University of Manitoba, Canada, was not involved in the collection of biological samples or the conduct of the experimental work. The samples were collected anonymously and no identifiable personal information was recorded. All subsequent experimental procedures were performed exclusively in vitro and involved no human intervention or clinical procedures. Because the study used anonymized biological material with no associated personal data and posed minimal risk to donors, the activity was conducted as a laboratory training exercise.

## Consent

Participation was voluntary and informed oral consent was obtained from all donors prior to saliva collection. Donors were informed of the purpose of the sample use and their right to decline participation. Samples were handled anonymously and no personal identifiers were recorded. The procedures followed the ethical principles for research involving human‐derived biological materials outlined in the 2013 revision of the Declaration of Helsinki.

## Conflicts of Interest

The authors declare no conflicts of interest.

## Supporting information


**Table S1:** Nutrition facts and ingredients of the food samples.

## Data Availability

All data supporting the findings of this study are provided within the manuscript and the Table S1.
