# Supplementary material for: Exploring the Influence of Oral‐Proximal Gastric in Vitro Digestion on Estimated Glycemic Index and Bioaccessibility of Bioactive Phenolic Compounds of Wheat‐Based Baked Products
Source: Food Sci Nutr. 2026 Jun 11;14(6):e72014. doi: 10.1002/fsn3.72014 (PMC13260707; doi:10.1002/fsn3.72014)
Supplement: Supplementary file 1 — Table S1: Nutrition facts and ingredients of the food samples. [file FSN3-14-e72014-s001.docx]

**Supplementary File**

**Exploring the Influence of Oral-Proximal Gastric *in vitro* Digestion on Estimated Glycaemic Index and Bioaccessibility of Bioactive Phenolic Compounds of Wheat Based Baked Products**

**Jasper Okoro Godwin Elechi *^1,2,3^, Diana Marisol Abrego-Guandique^4^, Roberto Cannataro^1,5,6^, Nicola Gasparre^2,3^, Erika Cione^1^**

^1^Department of Pharmacy, Health, and Nutritional Sciences. University of Calabria (Italy

^2^Department of Food and Human Nutritional Sciences. University of Manitoba (Canada)

^3^Richardson Centre for Food Technology and Research. University of Manitoba (Canada)

^4^ Department of Health Sciences, University of Magna Graecia Catanzaro, 88100 Catanzaro, Italy

^5^Galascreen Laboratories, University of Magna Graecia, 87036 Catanzaro, Italy

^6^Research Division, Dynamical Business & Science Society, DBSS International SAS, Bogota 110861, Colombia

*****✉ **Corresponding author:**
Jasper O.G. Elechi
Department of Pharmacy, Health and Nutritional Sciences, University of Calabria, 87036 Rende (CS), Italy
Email: [**jasper.elechi@ufpe.br**](mailto:jasper.elechi@ufpe.br)

**Supplementary Table S1 – NUTRITION FACTS & INGREDIENTS OF THE FOOD SAMPLES**

| 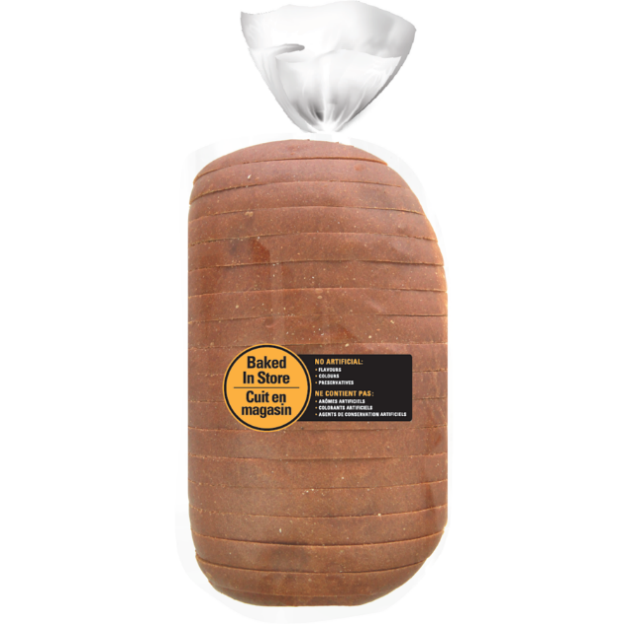**White Bread (WB)** | ***Nutritional Composition*** | ***Serving Size Per 75 g*** | ***% Daily Value**** | ***Ingredients*** |
| --- | --- | --- | --- | --- |
|  | **Calories**  **Fat**  Saturates  + Trans  **Carbohydrates**  Sugars  Fiber  **Protein**  Potassium  **Cholesterol**  **Sodium**  Vitamin A  Vitamin C  Calcium  Iron | 230 cal  5 g  1.5 g  0.0 g  40 g  4 g  2 g  7 g  0 mg  0 mg  360 mg  0.0  0.0  20 mg  2.25 mg | ---------  8 %  ---------  0 %  13 %  0 %  7 %  0 %  0 %  0 %  15 %  0 %  0 %  2 %  15 % | Enriched Wheat Flour, Water, Yeast, Sugar, Palm & Soybean Oil Shortening, Vegetable Oil, Whey Powder, Salt, Soy Lecithin, Enzymes, Ascorbic Acid. May Contain Peanuts, Tree Nuts, Sesame, Eggs &/or Other Allergen |
| 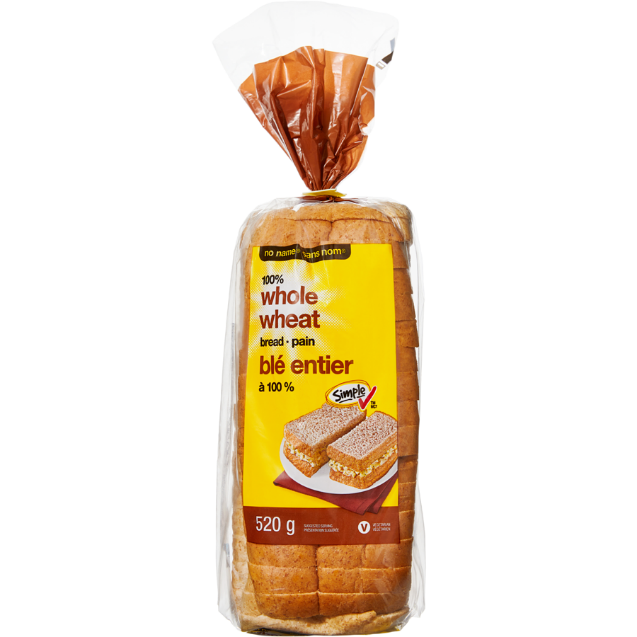  Whole Wheat Bread (WWB) | ***Nutritional Composition*** | ***Serving Size Per 58 g*** | ***% Daily Value**** | ***Ingredients*** |
|  | **Calories**  **Fat**  Saturates  + Trans  **Carbohydrate**  Sugars  Fiber  **Protein**  Potassium  **Cholesterol**  **Sodium**  Vitamin A  Vitamin C  Calcium  Iron | 140 cal  1.5 g  0.4 g  0.0 g  27 g  2 g  3 g  5 g  100 mg  0 mg  280 mg  -------  -------  40 mg  1.0 mg | ------  2 %  ------  ------  0 %  2 %  11 %  ------  2 %  ------  12 %  0 %  0 %  3 %  6 % | Whole Wheat Flour, Water, Yeast*, Sugar, Wheat Gluten*, vegetable Oil, Salt, Calcium Propionate, Sodium Stearoyl-lactate,vegetable Monoglycerides, Acetylated Tartaric Acid Esters Of Mono- And diglycerides, Sorbic Acid, Enzymes, Ascorbic Acid. *order May Vary. May Contain: Soy, Sesame. |
| 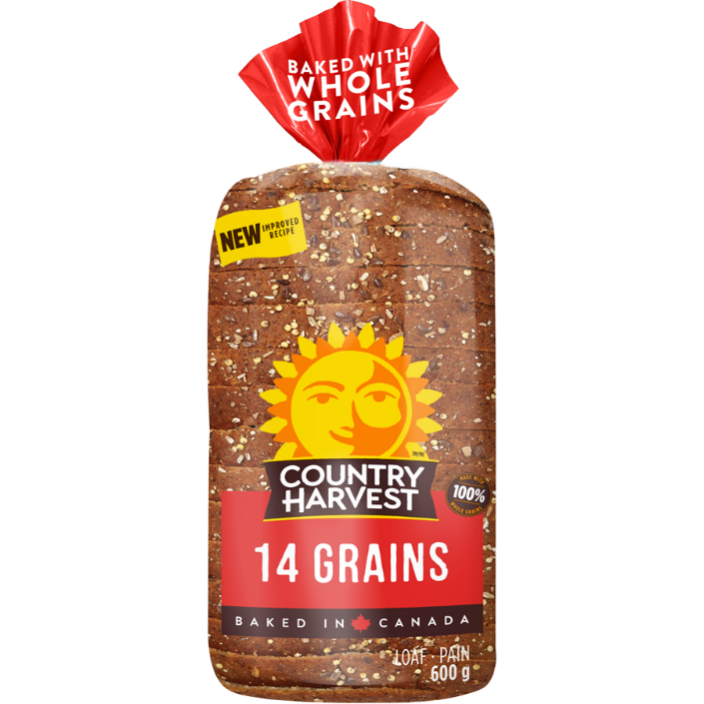**Multigrain bread (MGB) 14 Grain Bread** | ***Nutritional Composition*** | ***Serving Size Per 2.0 slices (80 g)*** | ***% Daily Value**** | ***Ingredients*** |
|  | **Calories**  **Fat**  Saturates  + Trans  Monounsaturates  Polyunsaturates  **Carbohydrate**  Sugars  Fiber  **Protein**  Potassium  **Cholesterol**  **Sodium**  Vitamin A  Vitamin C  Calcium  Iron  Thiamine  Riboflavin  Niacin  Vitamin B  Pantothenate  Phosphorus  Magnesium  Zinc  Selenium | 210 cal  3.0 g  0.5 g  0.0 g  1.0 g  1.5 g  38 g  5 g  6 g  10 g  200 mg  0 mg  310 mg  -------  -------  75 mg  2.0 mg  0.225 mg  0.175 mg  4.0 mg  60.175 mg  0.5 mg  175.0 mg  70.0 mg  1.75 mg  16.0 μg | --------  4 %  -------  4 %  ------  ------  0 %  5 %  21 %  ------  4 %  -----  13 %  0 %  0 %  6 %  11 %  19 %  13 %  25 %  10 %  10 %  14 %  17 %  16 %  29 % | Whole Grain Wheat Flour, Water, Whole Grain And Seed Mix (flax Seeds, Oat Flakes, Sunflower Seeds, Malted Crushed Wheat, Barley Flakes, Corn Meal, Rye Flakes, Triticale Flakes, Brown Rice Flour, Millet, Quinoa, Farro Flakes, Teff, Buckwheat Flour, Pumpkin Seeds), Sugar, Wheat Gluten*, Yeast*, Salt, Sour Starter (ground Wheat, Cultured Whole Grain Wheat Flour, Yeast, Lactic Acid [from Bacterial Culture]), Vegetable Oil, Calcium Propionate, Sorbic Acid, Soy Lecithin. Topping: Oat Flakes, Malted Crushed Wheat. *order May Vary. Contains: Wheat, Barley, Oats, Rye, Triticale, Soy. May Contain: Sesame Seeds. |
| 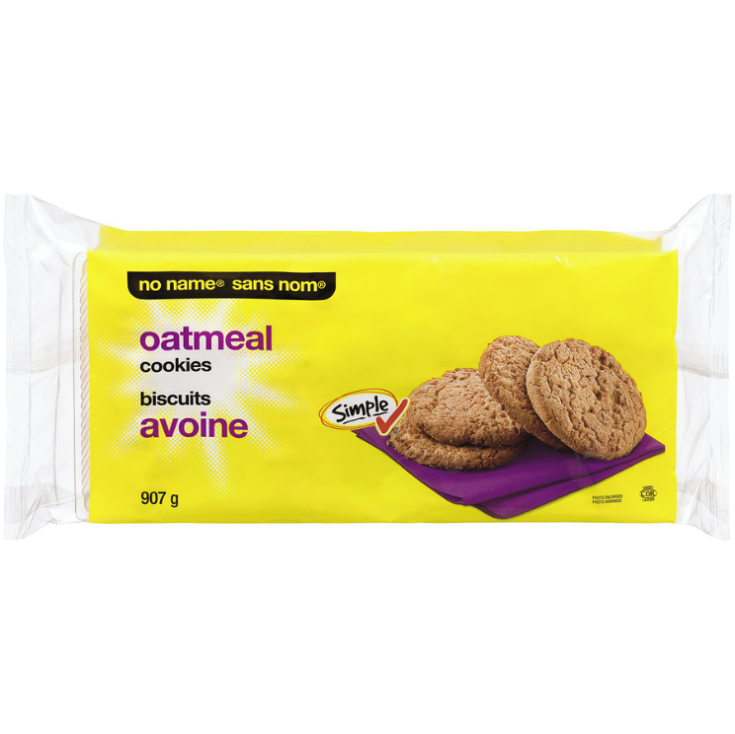  **Oatmeal Cookies (OMC)** | ***Nutritional Composition*** | ***Serving Size Per 33 g*** | ***% Daily Value**** | ***Ingredients*** |
|  | **Calories**  **Fat**  Saturates  + Trans  **Carbohydrate**  Sugars  Fiber  **Protein**  Potassium  **Cholesterol**  **Sodium**  Vitamin A  Vitamin C  Calcium  Iron | 150 cal  6 g  1.5 g  0.0 g  23 g  8 g  1 g  2 g  40 mg  0 mg  115 mg  ------  ------  10 mg  0.75 mg | 8 %  -----  -----  0 %  8 %  4 %  -----  1 %  -----  5 %  0 %  0 %  1 %  4 % | Enriched Wheat Flour, Sugars (sugar, Brown Sugar), Canola and Palm And Palm Kernel Oil Shortening, Rolled Oats, Water, Modified milk Ingredients, Baking Soda, Salt, Ammonium Bicarbonate, Natural flavour, Soy Lecithin, Dried Whole Egg, Caramel Colour, Maltol, Annatto, turmeric. May Contain: Peanuts, Tree Nuts. |

*5% or less is **a little**, 15% or more is **a lot**

The values stated are approximate and may not be fully representational of these products vitamins, nutrients and ingredients.
